# The Distribution of Bovine Tuberculosis in Cattle Farms Is Linked to Cattle Trade and Badger-Mediated Contact Networks in South-Western France, 2007–2015

**DOI:** 10.3389/fvets.2018.00173

**Published:** 2018-07-26

**Authors:** Malika Bouchez-Zacria, Aurélie Courcoul, Benoit Durand

**Affiliations:** ^1^Epidemiology Unit, Paris-Sud University, Laboratory for Animal Health, French Agency for Food Environment and Occupational Health and Safety (ANSES), Maisons-Alfort, France; ^2^Epidemiology Unit, Paris-Est University, Laboratory for Animal Health, French Agency for Food Environment and Occupational Health and Safety (ANSES), Maisons-Alfort, France

**Keywords:** bovine tuberculosis, network analysis, cattle herds, badger-cattle interface, cattle trade, pastures

## Abstract

Bovine tuberculosis (bTB), mainly caused by *Mycobacterium bovis*, can affect domestic and wild animals as well as humans. Identifying the major transmission mechanisms in an area is necessary *for* disease control and management. In this study, we aimed *to evaluate* the involvement of different types of contact in *M. bovis* transmission between cattle farms of south-western France between 2007 and 2015. We analyzed an empirical contact network of cattle farms as nodes, with known infection status and molecular types (16 circulated during the study period of which 14 affected only cattle and two both badgers and cattle). Edges were based on cattle trade data (T-edges) and on spatial neighborhood relationships between farms, either direct (P-edges) or badger-mediated, when two farms neighbored the same badger home range (B-edges), or two distinct but neighboring badger home ranges (D-edges). Edge types were aggregated so that the contact network contained only unique edges labeled by one or several edge types. The association between the contact network structure and bTB infection status was assessed using a non-parametric test, each molecular type being considered a marker of an independent epidemic. Using a logistic regression model, we estimated the contribution of each edge type to the probability for an edge originating from an infected farm to end at another infected farm. A total number of 1946 cattle farms were included in the study and were linked by 54,243 edges. Within this contact network, infected farms (whatever the molecular type) always belonged to the same component, suggesting the contact network may have supported bTB spread among those farms. A significant association between the pattern of bTB-infected farms and the structure of the contact network was observed when all the molecular types were simultaneously considered. The logistic regression model showed a significant association between *M. bovis* infection in direct neighbors of infected farms and the connection by T-, B- and D-edges, with odds-ratios of 7.4, 1.9, and 10.4, respectively. These results indicate a multifactorial *M. bovis* transmission between cattle farms of the studied area, with varying implication levels of the trade, pasture and badger networks according to the molecular type.

## Introduction

Since its discovery by Theobald Smith in the late 1800's (1) *Mycobacterium bovis*, the main agent of bovine tuberculosis (bTB) has been found in a wide variety of domestic and wild animal hosts, as well as in humans (2, 3). In Europe, the main host of *M. bovis* is cattle (4–6), but sheep (7), pigs (8) and goats (9) can be affected too. Wildlife species found infected on this continent include red deer (*Cervus elaphus*) (10, 11), roe deer (*Capreolus capreolus*) (12), red fox (*Vulpes vulpes*) (13–16), wild boar (*Sus scrofa*) (17, 18) and badger (*Meles meles*) (19–21).

Different routes may allow *M. bovis* transmission between wild and domestic hosts. The largest part of *M. bovis* shedding seems to occur through aerosols (respiratory tract secretions) and to a lesser extent through saliva, urine, feces (20, 22, 23), milk in cattle (24) and even wound exudates in badgers (20). Therefore close contacts (e.g., nose to nose) between infected individuals and susceptible ones can allow the transmission of *M. bovis*. However, several studies have shown that *M. bovis* may survive outside a host in a favorable environment for several months (24–26), allowing transmission through indirect contacts. *M. bovis* transmission between cattle can also involve different susceptible species either wild (27) or domestic [although the implication of other domestic species than cattle remains unclear regarding cattle transmission (24)]. At the herd level, several risk factors of bTB have been identified such as larger herd sizes, neighborhood with other herds, cattle movements, farm management practices such as grazing, dispersion of slurry on pastures or the share of water points (24, 28–31). Environmental risk factors have also been studied, with certain environmental conditions favoring the survival and persistence of *M. bovis* (such as shade, moisture or even some soil types) that foster *M. bovis* transmission (24–26). A third category of risk factors involves wildlife interactions, especially with badgers, wild boars and deer. For the latter two species, the sharing of feed or water on pastures appears to be a risk factor of *M*. *bovis* indirect transmission (23, 32, 33). The transmission between badgers and cattle seems a bit more complex, with uncertain direct contacts on pastures (34–36) and/or inside farm buildings (37). This interspecies transmission could occur on pastures through the shedding of the mycobacteria in urine and feces of infected badgers (24), and in respiratory tract secretions and feces of infected cattle (6, 29).

BTB molecular types are stable (38, 39) and can be used to trace independent epidemics (4). In France, while the officially bTB-free status was obtained in 2000, *M. bovis* infection has persisted in several regions. In 2014, 46% of incident outbreaks were detected in south-western France, with a national number of 105 cattle herds newly detected infected (40). Molecular typing methods spoligotyping (39) combined to MLVA (Multiple Loci Variable Number of Tandem Repeats, VNTR Analysis) based on MIRU-VNTR [Mycobacterial Interspersed Repetitive Unit–VNTR; (4, 38)] have allowed identifying 16 molecular types in this area between 2007 and 2015 from cattle isolates, two of which were shared between cattle and wildlife (4). Because spoligotype and MIRU-VNTR are considered stable markers (at least at a time horizon of several years), these 16 molecular types allow identifying 16 independent epidemics spreading in the same area during the same time period.

An effective way of representing the structure of contacts between hosts of an infectious disease consists in building networks (41), with epidemiological units as nodes, to which an infection status is associated. Edges linking nodes represent the contacts between epidemiological units that may allow the transmission of the disease agent. Regarding *M. bovis* transmission between cattle in France and in light of the above, nodes can represent cattle farms and edges may represent direct or indirect contacts between them. Two types of direct contacts may be featured by edges between farms: (i) contacts due to the trade of live cattle (42, 43) and (ii) contacts due to pasture neighborhood between cattle belonging to different farms but with nose to nose contacts over the fence (31, 44, 45). Besides, indirect contacts between cattle farms due the presence of wildlife may also be represented by edges. Concerning the badger, a known susceptible species to *M. bovis* infection (21, 40), the spatial organization of social groups with stable home ranges around setts (46, 47) allows us to represent indirect contacts with cattle based on the spatial intersection between farm pastures and home ranges (48).

The aim of our study was to analyze *M. bovis* transmission between cattle farms in a south-western area of France using contact networks and molecular types as infection status information. We built different networks featuring possible direct and indirect contacts between cattle farms and analyzed the association between their structure and the observed pattern of infected farms.

## Materials and methods

### Cattle data

**Figure 1:**
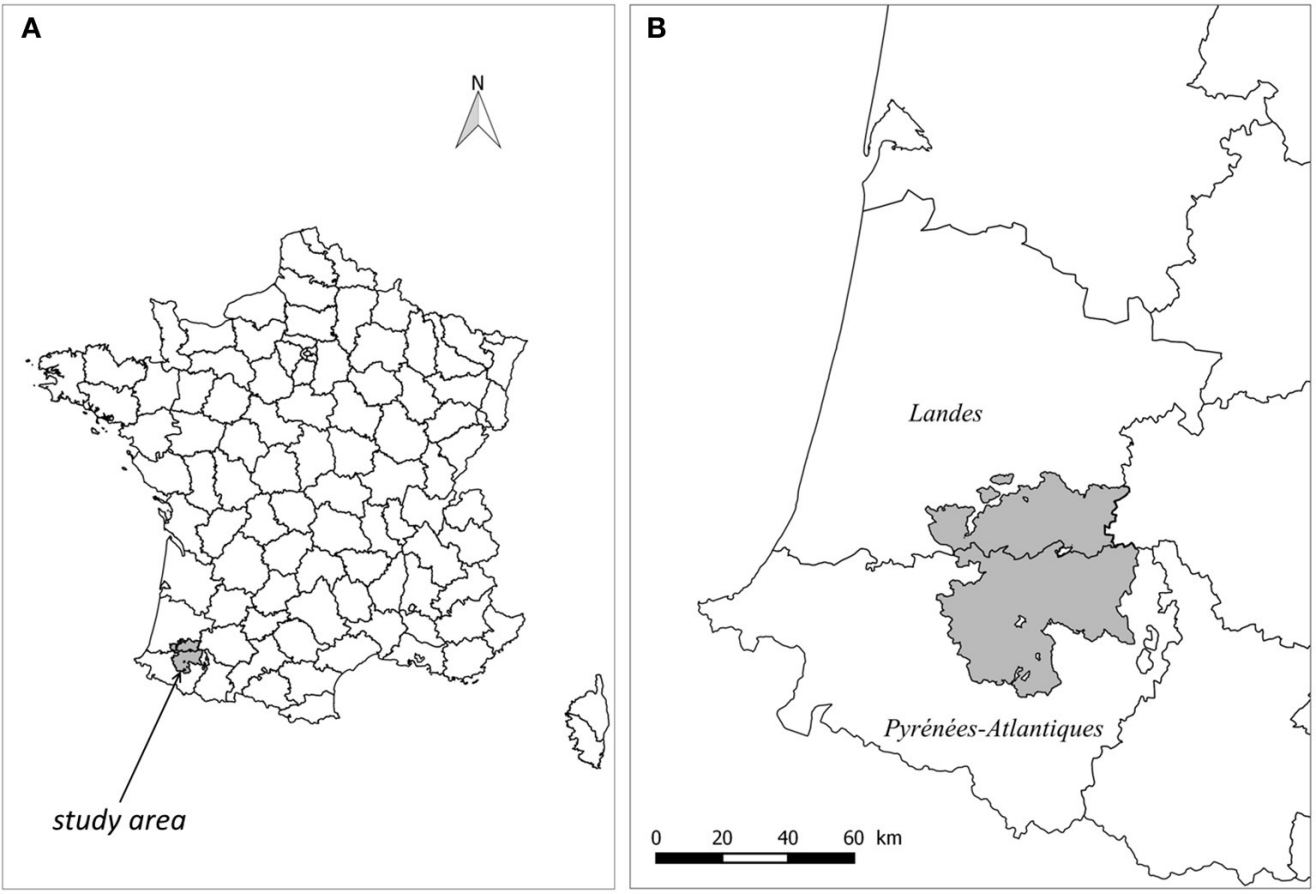
Location of the study area **(A)**, at the border between *Pyrénées-Atlantiques* (south) and *Landes* (north) French departments **(B)**.

The study population was made up of the 1946 farms having reported cattle between January 2007 and March 2016 (end of the 2015 herd skin-testing period) and owning at least one pasture included in a 2,735 km2 study area, an area straddling the border of *Pyrénées-Atlantiques* and *Landes* French departments (Figure 1). Pastures were defined as land parcels used by cattle for grazing according to the “*Relevé Parcellaire Graphique”* (RPG) of 2013 provided by the French Ministry of Agriculture. Two pastures were considered neighbors if the minimal distance between their borders was less than 3 m. Farm sizes (number of bovine females over two years old) and types (dairy, beef, fattening, mixed, small and other herds) were obtained from the French cattle tracing system (“*Base de Données Nationale d'Identification”* denoted below BDNI) (Table 1).

**Table 1:** Description of cattle farms included in the study population.

**Farm type**	**Number of farms**	Number of pastures (^*^)		Herd size (^**^)		Percentage of farms detected infected (^***^)
		**mean**	**SD**	**mean**	**SD**	
Beef	922	9.5	6.5	54.6	34.2	4.2 (*n* = 39)
Dairy	294	8.6	6.0	74.4	43.1	3.7 (*n* = 11)
Fattening	57	7.5	5.8	32.1	28.4	5.2 (*n* = 3)
Mixed	30	12.3	6.5	93.6	32.8	3.3 (*n* = 1)
Other	259	6.3	4.8	21.1	22.9	3.9 (*n* = 10)
Small	384	4.6	3.9	6.7	4.2	1.3 (*n* = 5)
All	1946	7.9	6.1	43.6	39.0	3.5 (*n* = 69)

* , pastures included in the study area;

** , number of females of more than 2 years old;

*** , at least once over the study period

BTB surveillance data were provided by the French Ministry of Agriculture. Herd skin-testing was performed each year in the study area in communes (the smallest French administrative subdivision) where infected farms had been detected the previous year, as well as in the neighboring ones, using either single intradermal comparative tuberculin tests (SICTT) (in all dairy farms or in farms located in the communes with confirmed infected farms) or single intradermal tuberculin tests (SITT) (in all the other situations), both performed in the cervical region. In the other communes of the study area, herd testing was biennial in *Landes* department, and triennial in *Pyrénées-Atlantiques* department. *M. bovis* infection was confirmed by polymerase chain reaction (PCR) and/or bacterial culture (either following a positive skin test or the detection of a suspect lesion during routine meat inspection at a slaughterhouse) (40) in 69 cattle farms of the study area during the study period; all the cattle of these farms were subsequently slaughtered and molecular typing was performed on each bovid found infected (with a mean of four cattle per farm detected infected during the study period). Molecular typing results were provided by the National Reference Laboratory (NRL) (Anses, Maisons-Alfort). The combination of spoligotyping and MLVA based on MIRU-VNTR allowed identifying 16 distinct molecular types (Table 2). A unique molecular type was identified in all of the 69 detected infected farms, except two where several molecular types were identified.

**Table 2:** Number of cattle farms detected infected per molecular type during the study period and within the study area.

**Molecular types**	**Number of farms**	**First and last year of detection**
SB0120b	1	2007
SB0120c	2	2009–2011
SB0121a	1	2012–2013^d^
SB0121b	1	2011
SB0121c^b^	1	2012
SB02065^b^	1	2012
SB0295^b^	1	2012
SB0821^a^^,^ ^c^	44	2007–2015
SB0823^c^	1	2010
SB0825^b^	1	2012
SB0827^b^	1	2012
SB0832^a^	13	2012–2015
SB0851	1	2011
SB0853	1	2009
SB0867^b^	1	2012
SB0928	4	2007–2012

a *molecular types found both in cattle and badgers*.

b *molecular types found in the farm where six molecular types were identified*.

c *molecular types found in the farm where two molecular types were identified*.

d *the same farm as in 2012 (recontamination)*.

A farm was classified infected by a given molecular type if this type had been detected at least once in the farm during the study period. Because of the geographic differences in the frequency of skin testing, having detected *M. bovis* earlier in a given farm than in another one does not imply that the former had been infected earlier than the latter. For this reason, the detection dates could not be taken into account.

### Badger data

Two thousand four hundred and 25 badger setts were identified and geolocalised by hunters in the study area, between 2013 and 2015. Around those setts, considered as main setts (i.e., hosting a social group), we defined badger home ranges using a two-step procedure: (i) a Dirichlet tessellation was first built around all setts [in which the perpendicular bisectors of each segment between two adjacent setts delineate the home range around one given sett, thus assuming that boundaries were located halfway between neighboring main setts (47)] and (ii) to avoid unrealistically home range large sizes, a home range was defined as the intersection of a tile with a 1,000 m-radius buffer area drawn around the setts (48). Two setts were considered neighbors if the corresponding home ranges were adjacent. A sett and a farm were considered neighbors if one of the farm pastures intersected with the badger home range.

BTB surveillance data were provided by the French Ministry of Agriculture. In the study area, bTB surveillance in badgers was performed according to the “Sylvatub” surveillance network, which started in 2012 in the study area (49). Surveillance protocol included badger trapping (i) within a 1.5 km-radius around confirmed infected farms, (ii) within a 2 km radius around setts with confirmed infected badgers and (iii) in communes at less than 5 km of communes where confirmed infected farms were located (one badger per sett). Trapping was performed using stopped restraints (https://www.plateforme-esa.fr/filedepot_download/35377/100) and snares were checked the morning after the day they were set up within the 2 h following sunrise, in order to limit the stress of trapped badgers. Trapped badgers were culled by head shot except in a minority of cases where they were found already dead (due to trap related injuries that sometimes occurred when snares were placed on sloping terrain, with no possible alternative). Road-killed badgers were also considered. Stopped restraints used for trapping were placed near sett entrances, those setts being considered as the sett of the trapped animals. Where badgers were found dead along roads, hunters reported the most probable sett according to their knowledge of the area (48). All the trapped and road-killed badgers were tested for *M. bovis* infection. Among 401 analyzed badgers (4.5% were road-killed badgers), 11.2% were detected infected (45 animals, one was a road-killed badger), of which 39 harbored the SB0821 molecular type and 6 the SB0832 molecular type, both molecular types having also been found in cattle (Table 2). All the badgers trapped could be attributed to 113 distinct setts, of which 33.6% hosted at least one infected badger (32 setts with at least one badger detected infected by SB0821 and 6 by SB0832). Road-killed badgers were attributed to five distinct setts. For four of these setts, the analysis of road-killed badgers did not provide additional information as they had also been subjected to trapping measures. For the fifth sett, the analysis of one road-killed badger allowed the detection of infection (SB0821 molecular type), not revealed by trapping. Setts with at least two badgers tested negative were considered as uninfected (*n* = 75). All the remaining setts, either with only one badger tested negative or without analyzed badger were considered of unknown status.

### Contact network

**Figure 2:**
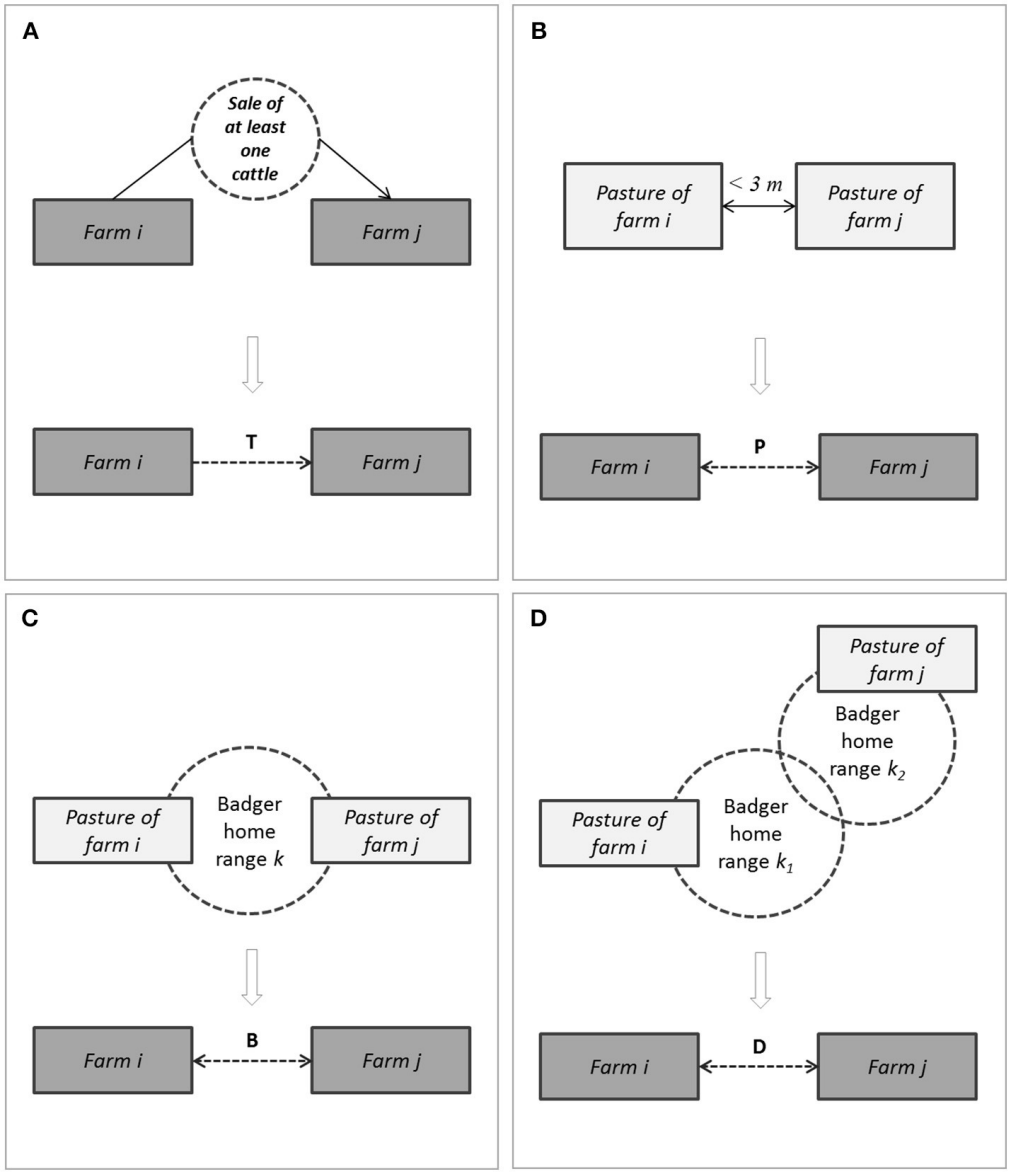
Schematic representation of the four types of edges between cattle farms in the contact network **(A)**, trade edge (noted T); **(B)**, pasture neighborhood edge (noted P); **(C)**, simple badger-mediated edge (noted B); **(D)**, second level badger-mediated edge (noted D).

A contact network was built using farms of the study population as nodes, and four types of edges (Figure 2):

- A trade edge (denoted T-edge below) from farms *i* to farm *j* represented the sale of one or several cattle by farm *i* to farm *j* during the study period, at one or several occasions;
- A pasture neighborhood edge (denoted P-edge below) between farms *i* and *j* represented the fact that a pasture owned by *i* and another one owned by *j* were neighbors;
- A simple badger-mediated edge (denoted B-edge below) between farms *i* and *j* represented the fact that both farms were neighbors of a given sett;
- A second level badger-mediated edge (denoted D-edge below) between farms *i* and *j* represented the fact that (i) farm *i* was neighbor of a sett *k*_1_, (ii) farm *j* was neighbor of a sett *k*_2_, and (iii) the setts *k*_1_ and *k*_2_ were themselves neighbors.

To avoid duplicated edges, the types of edges (T, P, B and D) were aggregated at the edge level. The full contact network thus contained only unique edges labeled by one or several edge types (Table 3). Because the T-edges are directed, each undirected P-, B- and D-edge was transformed into two symmetric directed edges. The full contact network was thus a directed network.

**Table 3:** Label of edges in the different networks of contacts between cattle farms in the study area.

**Edge label**	**Full contact network**	**T-network**	**P-network**	**B-network**	**D-network**	**Cattle-specific network**	**Badger-specific network**	**Mixed network**
**T**								
**P**								
**B**								
**D**								
**TP**								
**TB**								
**TD**								
**PB**								
**PD**								
**BD**								
**TPB**								
**TPD**								
**TBD**								
**PBD**								
**TPBD**								

*T, trade edge type; P, pasture neighborhood edge type; B, simple badger-mediated edge type; D, second level badger-mediated edge type; edge labels with several letters correspond to combinations of edge types; gray cells indicate the presence of the label within the network*.

Subnetworks were extracted from the full contact network by restricting the edges to those of specific types (Table 3). These subnetworks are termed below T-network, P-network, B-network and D-network. Similarly, we used edge types to split the full contact network in three non-overlapping subnetworks:

- the cattle-specific network incorporated edges labeled T, P or T-P, thus representing only contacts induced by cattle breeding practices;
- the badger-specific network incorporated edges labeled B, D or B-D, thus representing only badger-mediated contacts;
- the mixed network incorporated all the remaining edges, thus representing the co-occurrence of cattle-specific and badger-mediated contacts.

### Statistical analysis

Each of the 16 molecular types of *M. bovis* identified in the study area was considered as a marker of an independent epidemic. For a given molecular type, the contact network may be considered as supporting *M. bovis* transmission between two farms only if a path exists in the network between these farms. The transmission tree rooted on a detected infected farm should then be entirely located in a single component of the network. The contact network may then be considered as supporting the spread of a given molecular type if most of the farms infected by this molecular type are located in the same component of the contact network. We thus first computed, for each molecular type identified in more than one farm, the number of components in which these infected farms were located (50). For the same subset of molecular types, we also computed, for each infected farm, the length of the shortest path to another farm where the same molecular type was detected.

To evaluate whether the observed pattern of bTB infected farms may have resulted from transmission processes in the contact network, we used the *k*-test proposed by VanderWaal et al. (51). This permutation-based test is based upon the calculation of the *k*-statistic: the mean number of infected cases among the neighbors of an infected node (the approach is easily extended to neighborhoods of order >1). The observed value of this statistic is then compared to the distribution of the same statistic obtained by randomly reallocating the location of cases, thus simulating a possible pattern of cases under the null hypothesis of an absence of association between bTB case location and network structure. The empirical *p*-value of the *k*-test is then the proportion of permutations for which the *k*-statistic is greater than the observed one. We adapted this test to a multi-type epidemic by redefining the *k*-statistic as the mean number of cases among the neighbors of a node, which were infected by the same molecular type as that node.

The *k*-test was first performed on the full contact network. It was then applied on the cattle-specific, badger-specific and mixed subnetworks; and this, for two groups of molecular types: those observed in cattle only and those observed in cattle and in badgers. Seven tests were thus performed and the Bonferroni correction was applied. Ten thousand permutations were used to compute the empirical *p*-value.

To further analyse the association between edge types and bTB occurrence, we focused on edges originating from infected farms. A binary status was assigned to each of these edges, with a value of 1 when the destination node was infected by the same molecular type as the originating node, and 0 otherwise. The association between this status and the edge type was then assessed using a case-control design: cases were edges having a status of 1, and controls the edges having the status 0. Four binary explicative variables were defined, based on the types labeling the edge: T, P, B, and D. In addition, we took into account the size (number of bovine females over the age of 2 years) of the edge originating and destination farms, herd size being a well-known risk factor for bTB detection in cattle farms (24). We thus modeled the probability for an edge starting from a detected infected farm to end at a farm detected infected by the same molecular type, using a logistic regression model including six independent variables: four binary variables (presence/absence of the T, P, B and D edge type) and two quantitative variables (sizes of the originating and destination farms). We checked the absence of multicollinearity using variance inflation factors (VIF) with a threshold of 10 (52). Odds ratios (OR) and their associated 95% confidence intervals were computed. Finally, attributable risk fractions (AF) were computed for each edge type.

The definition of badger-mediated edges was based upon the neighborhood between pastures and one (B-edges) or two (D-edges) badger home ranges. For some of the corresponding setts, the trapping results allowed defining an infection status: setts were considered as (i) infected when at least one trapped badger had been found infected with an identified molecular type and (ii) uninfected when at least two trapped badgers had been tested negative and no occupant badger had been found infected [for more details, see (48)]. Based on these data, we finally used a Fisher exact test to analyze the association between the status of B- or/and D-edges and the infection status of the corresponding setts.

Dirichlet tessellations were computed using the deldir package (53) and buffers using the sp package (54). Network analyses were carried out using the igraph package (55) and variance inflation factors were computed using the car package (56). Attributable risk fractions were finally computed using the AF package (57). All those cited packages were used in R 3.3.2 (58).

## Results

**Figure 3:**
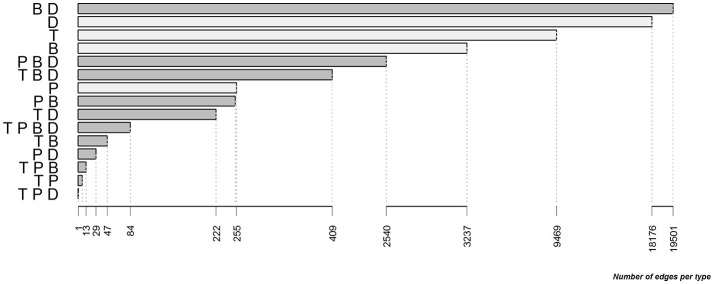
Distribution of the different types and combinations of types for the edges of the full contact network between cattle farms in the study area between 2007 and 2015 (T, trade edge; P, pasture neighborhood edge; B, simple badger-mediated edge; D, second level badger-mediated edge; edges having only one type are in light gray and combinations of several types are in gray).

Within the full contact network, the most frequent edge type was the combination of B- and D-edges, followed by single D-, T-, and B-edges. The P-edge type was less frequent alone than in combination with the other types (Figure 3).

The largest weak component of the full contact network incorporated 99.8% of the study population. Regarding the four edge-type-specific networks, the proportion of nodes included in the largest component was higher in trade and badger related networks (94.4% for the T-network, 94.7% for the B-network and 93.6% for the D-network) than in the pasture network (50.4%) (Table 4) (a more detailed analysis of networks topology is given in Supplementary Tables 2–4 and Supplementary Figures 1, 2).

**Table 4:** Description of the full contact network and of the four edge-type specific networks.

**Indicator**	**Full contact network**	***T*-network**	***P*-network**	**B-network**	**D-network**
*Number of nodes (size)*	1946	1946	1946	1946	1946
*Number of edges*	54243	10252	3182	26084	40962
*Number of components*	5	107	716	93	117
*Biggest component size*	1942	1837	980	1842	1822
*Second biggest component size*	1	2	23	6	4
*Number of components with one farm*	4	103	608	86	112

For each of the 16 molecular types, the farms where the type had been observed were always located in the same component of the full contact network. This was also the case for the B-network, but not for the T-, P-, and D- networks (Table 5).

**Table 5:** Distribution of detected infected farms in the components of the full contact network and in the four edge-type-specific networks for the molecular types identified in more than one farm.

	**Number of components containing detected infected farms**				
**Molecular types**	**Full contact network**	***T*-network**	***P*-network**	**B-network**	**D-network**
*SB0120c*	1	1	1	1	1
SB0821(^*^)	1	2	15	1	2
SB0832(^*^)	1	1	3	1	1
*SB0928*	1	1	3	1	1

* *, molecular types found both in badgers and cattle; see Table 2 for more details*.

**Figure 4:**
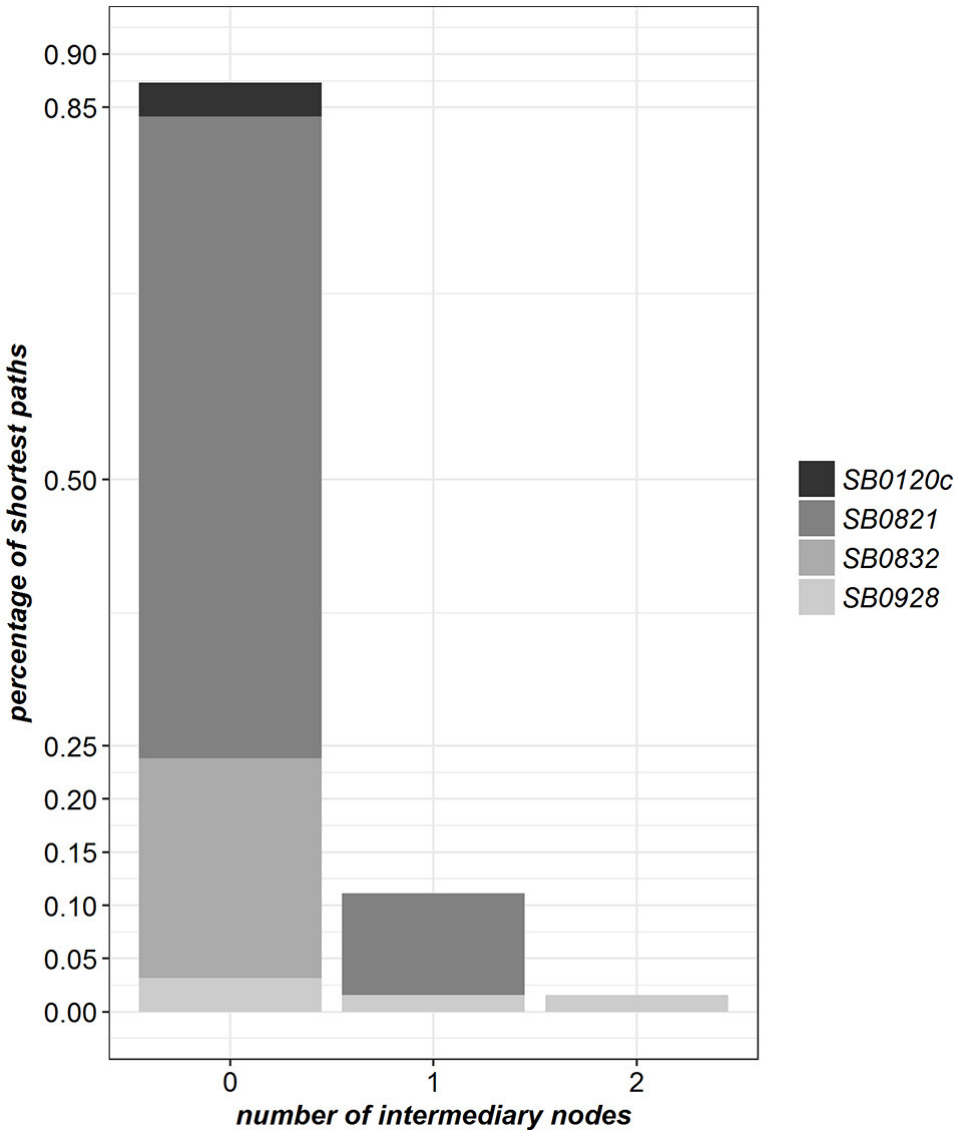
Distribution of the shortest path lengths in the full contact network between pairs of farms detected infected by the same molecular type (only the four molecular types found in at least two farms are considered).

Four molecular types were observed in at least two detected infected farms (Table 2). For 87% of these farms, the path to the closest farm detected infected by the same molecular type was made of a single edge. It included one intermediary cattle farm in 11% of cases (Figure 4 and Supplementary Table 2). This result suggests a prominence of *M. bovis* transmission between an infected farm and its direct neighbors in the full contact network.

We computed the proportion of shortest paths made of a single edge between farms infected (i) by molecular types found only in cattle and (ii) by molecular types found both in badgers and cattle. The difference between these two proportions was not significant (Fisher exact test: *p* = 0.13).

**Figure 5:**
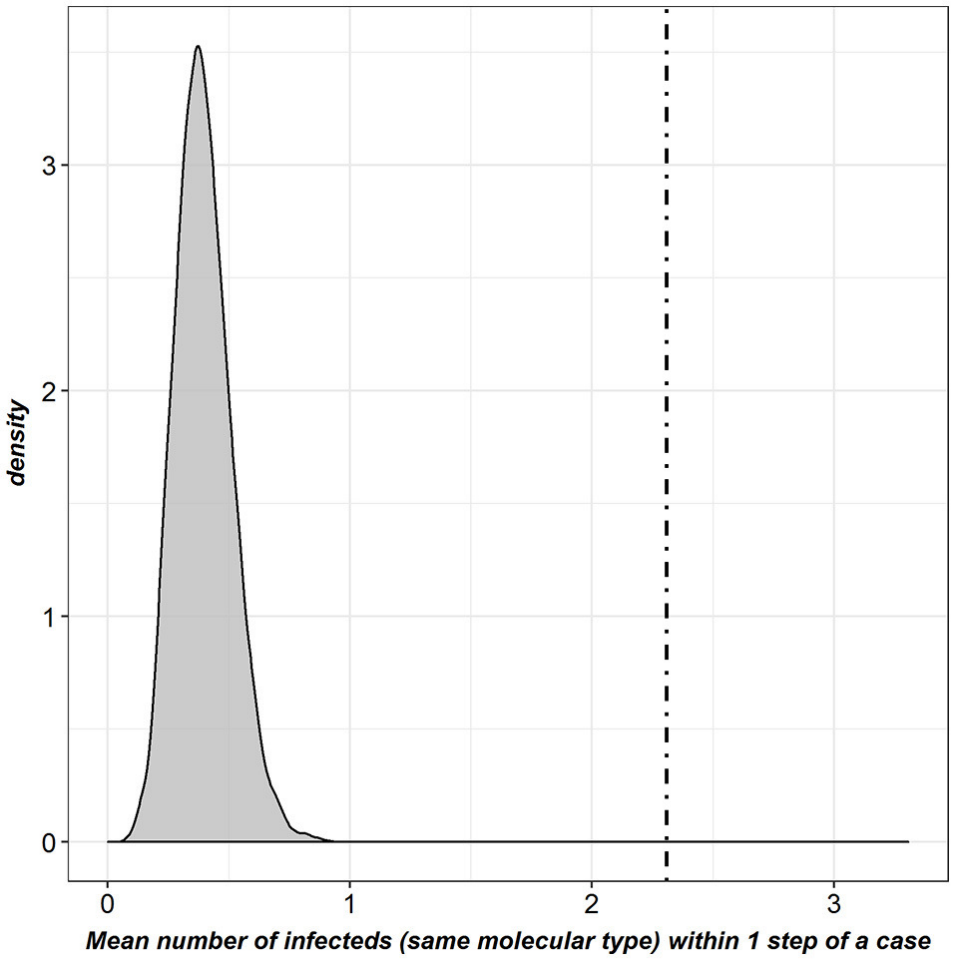
Graphical representation of the *k*-test results for the full contact network [*dot-dashed line: k*-statistic computed in the observed network; *gray density plot:* distribution obtained by randomly reallocating the location of cases; this last distribution was clearly lower than the *k*-statistic observed (*p* < 7.14^*^10^−3^, threshold after Bonferroni correction)].

Using *k*-tests, a significant association was observed between the pattern of bTB detected infected farms and the structure of the full contact network (observed *k*-statistic: 2.3; distribution obtained by randomly reallocating the location of cases: mean = 0.39, *SD* = 0.12; *p* < 7.14^*^10^−3^, threshold after Bonferroni correction) (Figure 5). No significant association was observed for the cattle-specific network, neither for the molecular types observed in cattle only, nor for those found both in cattle and badgers. Conversely, a significant association was observed between the pattern of farms detected infected by molecular types shared between badgers and cattle and the structure of the badger-specific network (*p* < 7.14^*^10^−3^). Finally, the structure of the mixed network was significantly associated with the pattern of bTB-infected farms for both groups of molecular types (*p* = 0.006 and *p* < 7.14^*^10^−3^ respectively) (Table 6).

**Table 6:** Results of the *k*-tests for the cattle-specific, the badger-specific and for the mixed subnetworks of the full contact network, for the molecular types only found in cattle only and for those found both in badgers and cattle.

			**Observed networks**	**Reallocated networks**	
**Molecular types found in**	**Networks**	***p*-value**	**k-statistic**	**Mean k-statistic**	**SD k-statistic**
Cattle only	Cattle-specific	1	0.00	0.002	0.01
	Badger-specific	0.07	0.11	0.008	0.03
	Mixed	0.006^*^	0.11	0.0008	0.01
Badger and cattle	Cattle-specific	0.027	0.23	0.09	0.06
	Badger-specific	0^*^	2.28	0.39	0.13
	Mixed	0^*^	0.46	0.03	0.03

* significant difference after Bonferroni correction (p < 7.14^*^10^−3^); SD, standard deviation.

The four edge types were included in the logistic regression model as no significant multicollinearity was detected. T-, B-, and D-edge types were significantly associated to the probability of being a case with an OR of 7.13 for the T-edge type (95% CI: [3.39–15.06]), 1.89 for the B-edge type (95% CI: [1.32–2.76]) and 10.44 for the D-edge type (95% IC: [4.38–26.66]). The size of the destination farm of the edge was also significantly associated to the probability of being a case. Regarding edge types, attributable risk fractions were 84% for the D edge type, 32% for the B edge type, and 12% for the T edge type (Table 7).

**Table 7:** Logistic model of the probability of an edge starting from a detected infected cattle farm to join another detected infected cattle farm and with the same molecular type according to the type of edge.

**Variable**	**Parameter estimate**	**OR (95% CI)**	***P*-value**	**AF (SD)**
*Intercept*	−5.02	0.01 [0.00–0.02]	<0.0001	-
*T edge type*	1.96	7.13 [3.39–15.06]	<0.0001	12% (6.2)
*P edge type*	0.30	1.35 [0.77–2.27]	0.26	3% (7.4)
*B edge type*	0.64	1.89 [1.32–2.76]	<0.0001	32% (8.6)
*D edge type*	2.34	10.44 [4.38–26.66]	<0.0001	84% (6.9)
*Size of the destination farm*	−0.0045	0.956 [0.910–0.996] (^*^)	0.049	-
*Size of the originating farm*	0.002	1.02 [0.99–1.06] (^*^)	0.19	-

OR, Odds ratio; CI, Confidence Interval; AF, Attributable Risk Fraction; SD, Standard Deviation. (

* *) Odds-ratio corresponding to an increase of ten animals*.

Among edges representing badger-mediated transmission (i.e., B- and D-edges), the infection status of badger setts involved (one sett regarding B-edges and at least one of the two setts regarding D-edges) was known for 264 edges (5%) originating from a farm infected by one of the two molecular types shared between badgers and cattle. Among them, 44 were case edges (i.e., the destination farm had also been found infected by the same molecular type) of which 38 (86%) were supported by positive badger setts; and 220 were control edges of which 102 were supported by positive badger setts (46%). These differences were significant (Fisher exact test: *p* < 0.0001) with an associated OR of 7.3 [95% CI: (2.9–21.9)].

## Discussion

The objective of this study was to provide a better understanding of *M. bovis* transmission mechanisms between cattle farms in south-western France using networks which represented the direct and indirect contacts that may allow *M. bovis* transmission among farms of this area between 2007 and 2015.

Four types of edges were represented because of their potential involvement in *M. bovis* transmission between cattle farms and we assumed that they represented the main transmission mechanisms in the study area. Cattle movements due to trade are a known *M. bovis* transmission route in Great Britain (59, 60), but also in France (42). The neighborhood with an infected farm through adjoining pastures (allowing over the fence contacts between herds) has also been identified as a potential risk factor for the *M. bovis* transmission between French cattle farms (31). The intersection of badger home ranges with cattle pastures and between each other's was considered a proxy for badger-mediated transmission, considering the territoriality of badgers (36) and the ability of *M. bovis* to survive in the soil (25, 26). BTB surveillance measures in badgers were not homogeneous among setts of the study area, as they were dependent on bTB detection in the cattle farms in their vicinity. For this reason, although the location of setts was known, we did not model badger setts as nodes in the contact network (we would have been unable to attribute an infection status to each of them). Instead of that, sett location data were used to represent badger-mediated contacts between farms by specific edges, based on neighboring badger home ranges. Two types of badger-mediated contacts were thus modeled by edges. B-edges represented a situation in which two farms neighbored the same badger home range: farm to farm *M. bovis* transmission through such edges thus only assumed cattle to badger and badger to cattle transmission. Conversely, D-edges represented a situation in which two farms neighbored two distinct but neighboring badger home ranges: farm to farm transmission through such edges thus also assumed badger to badger transmission in animals from neighboring setts. Because the epidemiological unit of this study was the farm, P-, B-, and D-edges were built based on the aggregation of pastures of each cattle farm. In the study area, cattle are often moved from one pasture to another one belonging to the same farm, e.g., when rotational grazing is used, we thus assumed that this simplification was meaningful.

The frequency of testing cattle was different in the different parts of the study area and this could have biased our results. However, testing was performed each year in communes where infected herds had been detected, and was also performed reactively in farms identified by contact tracing from these herds, based on cattle trade data and on pasture neighborhood. For these reasons, farms directly connected (in the full contact network) to a herd detected infected were considered having been submitted to similar testing regimens, both for B and D edge types (as in most cases the connected farms were located in the same commune), and for the T and P edge types (because of contact tracing). As only edges originating from herds detected infected were considered in the *k*-tests and in the logistic regression model, the corresponding results should not have been biased by geographic variations of the frequency of testing in the study area.

Taking into account the molecular types of isolates allowed considering 16 independent epidemics, of which 12 appeared restricted to a single farm, and 14 to less than 10 farms. All of these 14 molecular types affected only cattle. This predominance of molecular types found in a single cattle farm (75%) was in line with a previous study carried out in France between 1979 and 2000 in which a large majority of molecular types (84%) were found at a low frequency (less than 10 farms). This result has been interpreted as the sign of a poor spread of these strains (61), which could be traces of older epidemics that would have spread prior to 2007, but without significant transmission afterwards. Indeed, in our study, the 14 molecular types found in less than 10 farms were all detected not later than 2012 (Table 2).

Farms detected infected by a given molecular type were always located in the same large weak component of the full contact network that contained 99.8% of farms, whereas it was not the case for three of the edge-type-specific networks: the T-, P-, and D- networks. This indicated that, although the T-, P-, and D-edge-type-specific networks could not alone have supported the spread of bTB infection within the study area (contrary to the B-network), the strong connectivity resulting from the union of the four networks into the full contact network provided a structure that might enable the spread of the *M. bovis* infection in the study area. This result is in line with multifactorial mechanisms of bTB spread previously suggested by other studies (24, 29). As an example in Great Britain, dynamic modeling of cattle taking into account farm environment helped understanding *M. bovis* transmission routes (62). Prominent identified routes of *M. bovis* transmission were moving infected cattle between farms and reinfection from an environmental reservoir. The conclusion of this study was that control measures should simultaneously address several transmission routes to be effective.

Using *k*-tests, a significant association was observed between the pattern of bTB-infected farms and the structure of the full contact network. Moreover, the structures of the badger-specific and mixed networks were significantly associated with the pattern of farms detected infected by molecular types shared between badgers and cattle. This result was expected and confirmed that badger-mediated edges could be viewed as paths for the interspecies *M. bovis* transmission. In addition, the structure of the mixed network was significantly associated with the pattern of bTB-infected farms for molecular types found only in cattle, whereas it was not the case for the cattle-specific network. We could assume that the spread of cattle molecular types would be more efficient when direct contact (trade and/or pasture neighborhood) are associated with indirect badger-mediated contacts. In addition, we should be cautious about the cattle specificity of these molecular types, as these molecular types may be (or have been) present in the badger population without being observed, because of the relatively low sensitivity of bTB surveillance in the badger population.

Considering edges originating from detected infected farms, we used a case-control design and a logistic model to analyse the relationship between the types of an edge and the detection of the same molecular type at the originating and destination farm of the edge (case edges) or at the originating farm only (control edges). Because the detection dates could not be considered in the study to infer dates of infection, the co-occurrence of the same molecular type at both ends of case edges does not model the transmission of *M. bovis* through the edge, although the edges of the full contact network represent possible transmission paths for the bacteria and case edges thus represent possible transmission events. The largest odds-ratio was attributed to the D edge type, followed by the T edge type. This predominance of badger-mediated edges reflects the specific situation of the study area, where molecular types shared between badgers and cattle were predominant (84% of detected infected farms Table 2), and the predominant effect of the D edge type suggests a probable spread of *M. bovis* between badgers from neighboring setts, and not only between badgers and cattle. However, B and D edges were defined based on a geographic representation of home ranges, with a maximal distance of 1,000 m to the sett. This distance threshold, the Dirichlet tessellation used to model home ranges, and the fact that some setts may have been unoccupied, are three elements that may have led to an underestimation of home range size, and to an overestimation of the role of the D edge type.

The T edge type was also associated with a putative transmission of *M. bovis* (*AF* = 12%). This result is in agreement with a previous French study conducted at the national scale, according to which the population attributable risk fraction of bTB infection had been estimated at 12% [5–18%] for cattle trade (42), often allowing long distance bTB spread.

In a previous study conducted in France, pasture neighborhood was found significantly associated with the farm infection status (31). However, in the present study, the P edge type was not significantly associated with *M. bovis* transmission when using the case-control design. This may be first explained by the fact that some of farmers of the study area use rotational grazing, with some pastures left unoccupied for grass re-growth. Furthermore, P-edges were defined based on a direct neighborhood between pastures (<3 m). This short distance does not allow other opportunities of direct contacts between cattle, such as the wandering of livestock, to be represented.

The badger-specific edges (B and D edge types) were defined based on sett locations, one or two setts being associated to each. For some of these setts, an infection status could be determined based on bTB surveillance data. We showed that this infection status was significantly associated with the fact that the sett as well as the originating and the destination farms had all been found infected by isolates of the same molecular type (*OR* = 7.3; 95% CI:[2.9-21.9]). This result supports an actual badger-mediated transmission through these types of edges. Nevertheless, wild boars have also been found infected with *M. bovis* within the study area. Indeed, among 548 analyzed wild boars between 2011 and 2015, 15 (2.7%) were found infected. The corresponding molecular types found in these wild boars were the two molecular types shared between badgers and cattle. Therefore we cannot exclude the role of this wild species that we could not consider in this study because of a lack of field data that would have allowed its spatial organization (captured through radio tracking, for example) to be represented. Not considering wild boars in our analyses could have led to an over-estimate of the role of B and D edge types in *M. bovis* transmission between cattle farms.

Other indirect contacts through herd practices could also have contributed to the predominance of the D edge type. Indeed, this type of edge created links between farms without direct contacts at pasture but being in a kind of vicinity. As examples, the sharing of material or the loan of animals could create links between farms that may overlap the D edges. However, no data were available to investigate this assumption. Its confirmation or refutation would require supplementary investigation.

In conclusion, this study supports the multifactorial nature of *M. bovis* transmission between cattle farms within the *Pyrénées-Atlantiques–Landes* area, France from 2007 to 2015. The largest part of bTB spread seemed to be due to badger-mediated contacts, however cattle trade played a significant role. Consequently, to be truly effective, control measures should not focus on a single type of contact but ought to act on the different mechanisms we raised.

## Author contributions

MB-Z, AC, and BD conceived and designed the study. MB-Z prepared the data for the analysis. MB-Z and BD performed the analysis. MB-Z wrote the manuscript. MB-Z, AC, and BD revised the manuscript. All the authors approved the submitted version of the manuscript.

### Conflict of interest statement

The authors declare that the research was conducted in the absence of any commercial or financial relationships that could be construed as a potential conflict of interest.
