# Supplementary material for: The Distribution of Bovine Tuberculosis in Cattle Farms Is Linked to Cattle Trade and Badger-Mediated Contact Networks in South-Western France, 2007–2015
Source: Front Vet Sci. 2018 Jul 26;5:173. doi: 10.3389/fvets.2018.00173 (PMC6071575; doi:10.3389/fvets.2018.00173)
Supplement: Supplementary file 1 [file Data_Sheet_1.docx]

Supplementary Material

The distribution of bovine tuberculosis in cattle farms is linked to cattle trade and badger-mediated contact networks in south-western France, 2007-2015

Malika Bouchez-Zacria, Aurélie Courcoul, Benoit Durand*

*** Correspondence:** Benoit Durand: [benoit.durand@anses.fr](mailto:benoit.durand@anses.fr)

# Descriptive analysis of full contact network and specific networks: methods

*Indicators*

Social network analysis methods were used to describe the full contact network, as well as the four edge-type-specific networks (i.e. T-, P-, B- and D-networks), using several network and node indicators for each network. The network indicators we computed were the number of nodes, the number of edges, the diameter, the density and the average path length. The node indicators we computed were the mean degree, the assortativity, the mean betweenness and the clustering coefficient (Supplementary Table 1).

*Modularity*

The sizes of the two largest weak components (1,2) were computed, as well as the number of components with only one node (Supplementary Table 1).

Supplementary Table 1: Definition of network, modularity and node indicators computed in the study

|  |  | **Denomination** |  | **Definition in the study** |
| --- | --- | --- | --- | --- |
| **NETWORK** | **Indicators** | *Number of nodes or size* |  | The number of cattle farms included in the network (in our study all nodes are cattle farms) |
|  |  | *Number of edges* |  | The number of links between cattle farms within the network |
|  |  | *Diameter* |  | The longest distance (*i.e.* number of edges, without repeated edges, along the shortest path connecting them) between two cattle farms among all distances between any pair of cattle farms (3) |
|  |  | *Density* |  | The number of edges observed within the network among the number of all possible edges between two cattle farms of the network (1) |
|  |  | *Average path-length* |  | Mean distance between two cattle farms, averaged across all pairs of cattle farms (3) |
|  | **Modularity** | *Number of components* |  | The number of maximally connected subregions of a network in which all pairs of cattle farms are linked (directly in our study, each edge defined with an origin and a receiver). In the study, we considered weak components, i.e. areas of the network in which cattle farms are linked but ignoring the direction of the edge (2). |
|  |  | *Biggest component size* |  | The component with the maximal number of cattle farms (1) |
|  |  | *Second biggest component size* |  | The component with the second maximal number of cattle farms (1) |
|  |  | *Number of components with one farm* |  | The number of components with only one cattle farm |
| **NODES** | **Indicators** | *Average degree* |  | Mean of all the degrees of cattle farms within the network. The degree of a node (*i.e.* a cattle farm here) is the number of connections of a cattle farm to other cattle farms within the network (3) |
|  |  | *Assortativity* |  | Correlation level between the degree of one cattle farm and its neighbors (*i.e.* with which it is directly connected) within the network (4,5) |
|  |  | *Average betweenness* |  | Mean of all betweennesses of all cattle farms within the network. The betweenness of a node (*i.e.* a cattle farm here) is the number of shortest path lengths that pass through the node (1). |
|  |  | *Clustering coefficient* |  | The average fraction of pairs of neighbors of a cattle farm that are also neighbors of each other (3) |

*Topology*

To assess the structure type of networks we built, we generated 100 random networks with the same number of nodes and computed the distribution of their average path-length and clustering coefficient. According to (1), a clustering coefficient more than 20 times greater than a similar average path-length calculated on random networks would indicate a small-world topology of the studied network. In such networks, an infection could spread not only locally within groups (clusters) of cattle farms, but also to groups (clusters) that are topologically distant in the network (6). The log-log distribution of degrees throughout the nodes of the network is also a well-known way to characterize complex real networks. Indeed, in random networks, the number of nodes with high degrees decline exponentially. This is not the case in scale-free networks for which a linear trend can be observed in tail of the degree distribution (with a log-log scale), due to a power-law distribution of degrees declining more slowly than in random networks (3). Such a topology would allow an infection to spread faster than in random networks of a similar size, due to the presence of highly connected nodes (6).

# Descriptive analysis of full contact network and specific networks: results

*Indicators*

The full contact network as well as all specific networks incorporated 1946 cattle farms. Among the four specific networks, those farms were the least connected within the P-network (3182 edges) and the most connected within the B-network (40962 edges). In line with this, the densities of these networks were respectively the lowest and the highest, and conversely for their diameters and average path lengths. The number of neighbors for a cattle farm within the badger-mediated networks was higher than within the cattle contacts related networks (the average degree of the B- network was more than two times higher than the T-network and more than four times higher than the D-network). The correlation between highly connected cattle farms was the highest within the B-network (assortativity: 0.23). The average betweennesses of all networks were quite similar, revealing that even in the lowest connected networks as the P-network, a cattle farm could be represented on paths between several other connected groups (Supplementary Table 2).

Supplementary Table 2: Indicators computed for the contact and the four specific networks

|  |  | **Indicator** | **Full contact network** | **T-network** | **P-network** | **B-network** | **D-network** |
| --- | --- | --- | --- | --- | --- | --- | --- |
| **NETWORK** | **Indicators** | *Number of nodes (size)* | 1946 | 1946 | 1946 | 1946 | 1946 |
|  |  | *Number of edges* | 54243 | 10252 | 3182 | 26084 | 40962 |
|  |  | *Diameter* | 9 | 13 | 33 | 16 | 12 |
|  |  | *Density (*10^-3^)* | 14.33 | 2.71 | 0.84 | 6.89 | 10.82 |
|  |  | *Average path-length* | 3.10 | 5.02 | 13.18 | 5.15 | 4.28 |
|  | **Modularity** | *Number of components* | 5 | 107 | 716 | 93 | 117 |
|  |  | *Biggest component size* | 1942 | 1837 | 980 | 1842 | 1822 |
|  |  | *Second biggest component size* | 1 | 2 | 23 | 6 | 4 |
|  |  | *Number of components with one farm* | 4 | 103 | 608 | 86 | 112 |
| **NODES** | **Indicators** | *Average degree*  *[2.5^th^-97.5^th^ percentile]* | 55.75  [6-144.37] | 10.54  [0-47] | 3.27  [0-12] | 26.81  [0-78] | 42.10  [0-118] |
|  |  | *Assortativity* | 0.06 | -0.05 | 0.13 | 0.23 | 0.176 |
|  |  | *Average betweenness*  *[2.5^th^-97.5^th^ percentile]* | 1.07*10^-3^  [2.88*10^-6^-5.81*10^-3^] | 1.06*10^-3^  [0-7.65*10^-3^] | 1.59*10^-3^  [0-1.74*10^-2^] | 1.91*10^-3^  [0-1.52*10^-2^] | 1.48*10^-3^  [0-1.20*10^-2^] |
|  |  | *Clustering coefficient* | 0.26 | 0.06 | 0.14 | 0.42 | 0.43 |

The same indicators were computed in order to describe the cattle-specific, the badger-specific and the mixed networks. These three sub-graphs included the 1946 cattle farms with respectively 9731, 40 914 and 3598 edges between them. With the highest density, the badger-specific network was the most connected. The percentages of cattle farms included in the biggest component were respectively 93.42%, 95.22% and 64.7%. The percentages of components including only one farm were respectively 97.62%, 93.98% and 87.66% (Supplementary Table 3).

**Supplementary Table 3:** Indicators computed for the cattle-specific, the badger-specific and the mixed networks

|  |  |  | **Networks** | | |  |
| --- | --- | --- | --- | --- | --- | --- |
|  |  | **Indicator** | **Cattle-specific** | **Badger-specific** | **mixed** | |
| **NETWORK** | **Indicators** | *Number of nodes or size* | 1946 | 1946 | 1946 | |
|  |  | *Number of edges* | 9731 | 40914 | 3598 | |
|  |  | *Diameter* | 13 | 12 | 27 | |
|  |  | *Density (*10^-3^)* | 2.571 | 10.81 | 0.951 | |
|  |  | *Average path-length* | 5.206 | 4.258 | 11.29 | |
|  | **Modularity** | *Number of components* | 126 | 83 | 559 | |
|  |  | *Biggest component size* | 1818 | 1853 | 1259 | |
|  |  | *Second biggest component size* | 3 | 6 | 11 | |
|  |  | *Number of components with one farm* | 123 | 78 | 490 | |
| **NODES** | **Indicators** | *Average degree*  *[2.5^th^-97.5^th^ percentile]* | 10.00  [0-45.37] | 42.05  [0-113.37] | 3.70  [0 -14.00] | |
|  |  | *Assortativity* | -0.03 | 0.17 | 0.12 | |
|  |  | *Average betweenness*  *[2.5^th^-97.5^th^ percentile]* | 1.11*10^-3^  [0-8.60*10^-3^] | 1.52*10^-3^  [0-12.46*10^-3^] | 1.76*10^-3^  [0-14.65*10^-3^] | |
|  |  | *Clustering coefficient* | 0.05 | 0.40 | 0.15 | |

*Topology*

The P- and B- networks showed a small-world topology: (i) their clustering coefficients were more than 20 times higher than the mean clustering coefficients of the 100 random networks we generated and (ii) their average path-lengths were similar to these last ones (Supplementary Table 4). Thus, neighbors of a given cattle farms within those networks had a high probability to be themselves neighbors, and most cattle farms could be in contact with another cattle farm through a low number of steps.

The log-log distribution of degrees showed a linear trend for the T-network (Supplementary Figure 1) and indicated a scale-free structure for this network. This structure is said to be polarized, with few cattle farms highly connected to other cattle farms themselves weakly connected.

**Supplementary Table 4:** Comparison of the clustering coefficients, average path-lengths and diameters between networks and 100 random networks

| **Network** | **contact** | **T** | **P** | **B** | **D** |
| --- | --- | --- | --- | --- | --- |
| *Clustering coefficient (observed)* | 0.256 | 0.06 | 0.145 | 0.419 | 0.433 |
| *Mean clustering coefficient (random)*  *[2.5^th^-97.5^th^percentile]* | 0.028  [0.028 – 0.029] | 0.005  [0.005 - 0.006] | 0.002  [0.001 – 0.003] | 0.014  [0.013 – 0.014] | 0.022  [0.021 – 0.022] |
| *Average path-length (observed)* | 3.101 | 5.024 | 13.182 | 5.151 | 4.279 |
| *Mean average path-length (random)*  *[2.5^th^-97.5^th^percentile]* | 2.647  [2.647 – 2.648] | 4.74  [4.721 – 4.758] | 12.833  [12.116 – 13.53] | 3.194  [3.192 – 3.197] | 2.793  [2.792 – 2.794] |
| *Diameter (observed)* | 9 | 13 | 33 | 16 | 12 |
| *Mean diameter (random)*  *[2.5^th^-97.5^th^percentile]* | 4  [4 – 4] | 9.58  [9 – 10] | 34.63  [30 – 40.525] | 5.02  [5 – 5] | 4  [4 – 4] |


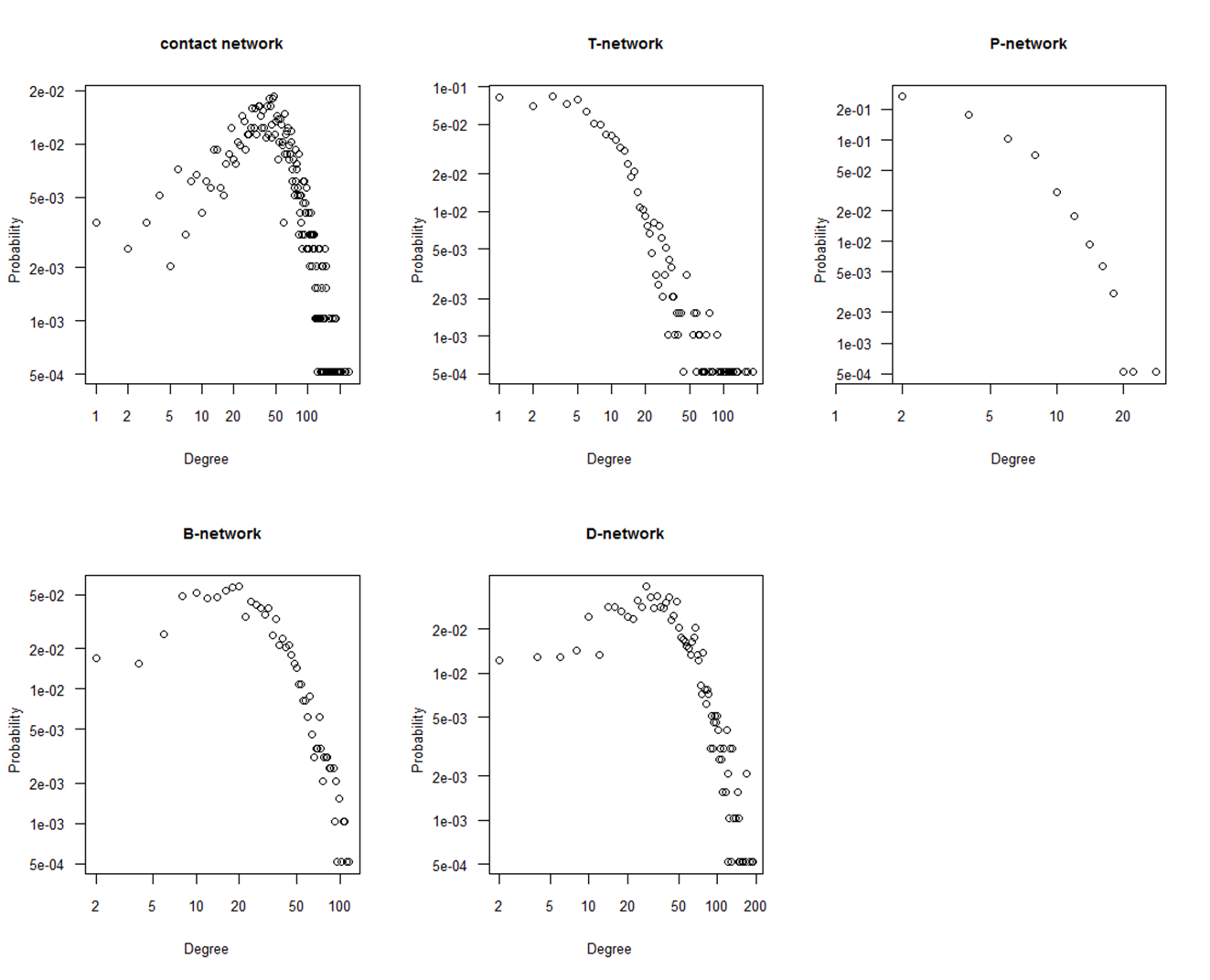


Supplementary Figure 1: Log-log distribution of degrees for the contact and the edge-type specific networks

Within each edge-specific network, several combinations of types were found. While the T-edge types were predominant within the T-network, the combinations of P- and badger-mediated types were more frequent than the single P-edge type within the P-network. Within both B- and D-networks, the combination of the two badger networks types was more frequent than the single badger related type (Supplementary Figure 2).


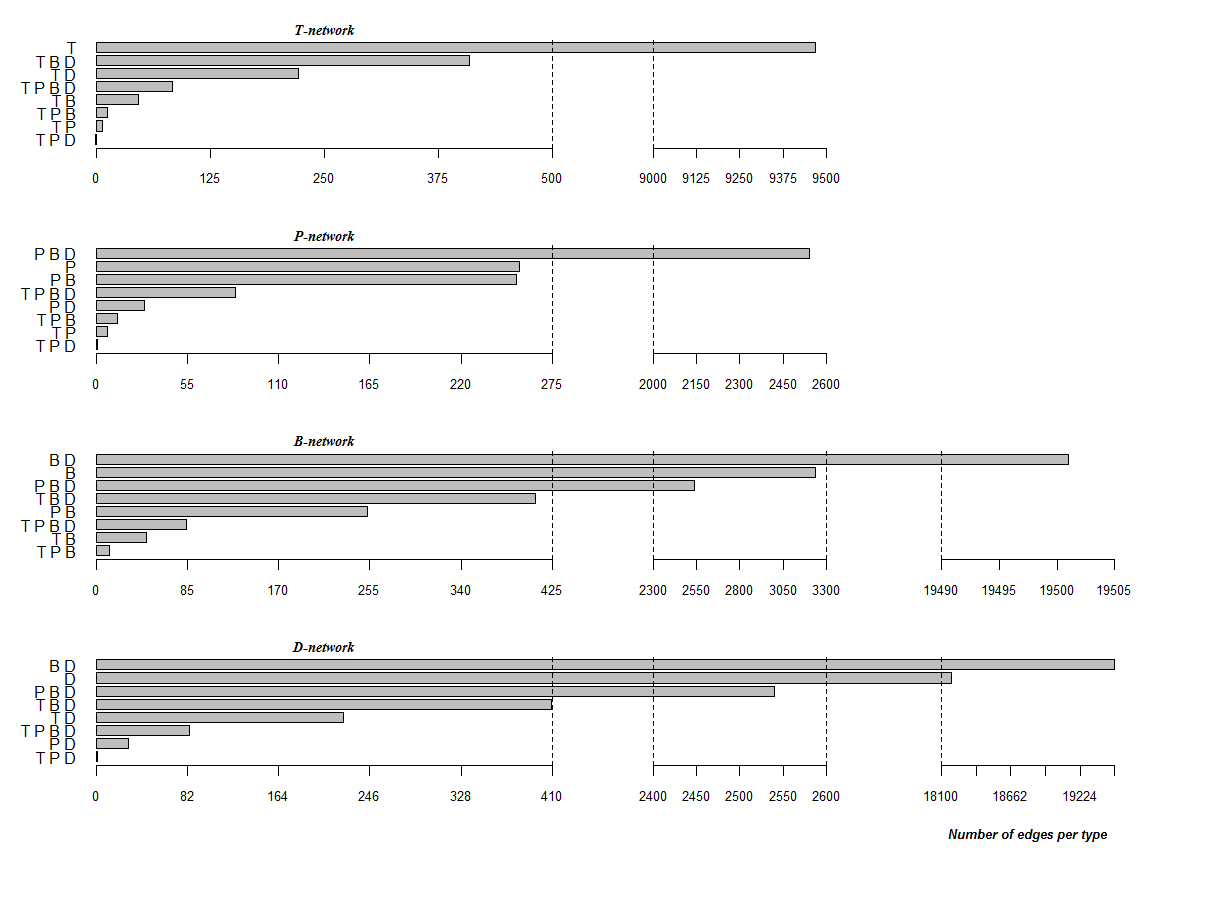


**Supplementary Figure 2:** Distribution of the different types and combinations of types for edges within each edge-specific network (T: T-edge type; P: P-edge type; B: B-edge type; D: D-edge type)

**References**

1. Dubé C, Ribble C, Kelton D, McNab B. Introduction to network analysis and its implications for animal disease modelling. *Rev Sci Tech Int Off Epizoot* (2011) **30**:425–436. doi:https://doi.org/10.20506/rst.30.2.2043

2. Robinson SE, Everett MG, Christley RM. Recent network evolution increases the potential for large epidemics in the British cattle population. *J R Soc Interface* (2007) **4**:669–674. doi:10.1098/rsif.2007.0214

3. Wang XF, Chen G. Complex networks: small-world, scale-free and beyond. *IEEE Circuits Syst Mag* (2003) **3**:6–20. doi:10.1109/MCAS.2003.1228503

4. Csardi G, Nepusz T. *The igraph software package for complex network research.* InterJournal Complex Systems, 1695, 1–9 (2006).

5. Newman MEJ. Assortative mixing in networks. *Phys Rev Lett* (2002) **89**: doi:10.1103/PhysRevLett.89.208701

6. Dubé C, Ribble C, Kelton D, McNab B. Estimating potential epidemic size following introduction of a long-incubation disease in scale-free connected networks of milking-cow movements in Ontario, Canada. *Prev Vet Med* (2011) **99**:102–111. doi:10.1016/j.prevetmed.2011.01.013
